# Early Negativization of SARS-CoV-2 Infection by Nasal Spray of Seawater plus Additives: The RENAISSANCE Open-Label Controlled Clinical Trial

**DOI:** 10.3390/pharmaceutics14112502

**Published:** 2022-11-18

**Authors:** Luca Cegolon, Giuseppe Mastrangelo, Enzo Emanuelli, Riccardo Camerotto, Giacomo Spinato, Daniele Frezza

**Affiliations:** 1 Department of Medical, Surgical & Health Sciences, University of Trieste, 34137 Trieste, Italy; 2 Occupational Medicine Unit, University Health Agency Giuliano Isontina (ASUGI), 34148 Trieste, Italy; 3 University of Padua, 35122 Padova, Italy; 4 Otolaringology Unit, Ca’ Foncello Hospital, Local Health Unit N.2 “Marca Trevigiana”, 31100 Treviso, Italy; 5 Section of Otolaringology, Department of Neurosciences, University of Padua, 35122 Padua, Italy

**Keywords:** SARS-CoV-2, COVID-19, nasal irrigation, hypertonic saline solution, viral shedding time, early negativization, swab test

## Abstract

**Background**: COVID-19 is an asymptomatic condition in 40% of cases, and most symptomatic patients present with mild/moderate disease not requiring hospitalization or intensive care, especially during the Omicron wave, when the hospitalization rate was estimated to be 0.3%. The main port of entry for SARS-CoV-2 in the human body is the nasal cavity and the upper respiratory tract is affected since the early stages of the infection. Nasal irrigation or aerosol by isotonic or hypertonic saline solution is a traditional therapeutic approach for respiratory or nasal inflammation, also featured by prophylactic properties against upper respiratory infections. **Methods**: We conducted a prospective open-label controlled study to assess the superiority of an already existing medication (Tonimer Lab Panthexyl 800)—a sterile hypertonic solution containing seawater, xylitol, panthenol and lactic acid—to reduce the viral shedding time in patients affected by asymptomatic or mild COVID-19. COVID-19 patients (N = 108) were split into two groups: a treatment arm (50 participants receiving standard of care plus nasal spray 3 times/day with Tonimer Lab Panthexyl 800) and a control arm (58 participants receiving standard of care but nasal spray with Tonimer Lab Panthexyl 800). The two groups, both testing initially positive for SARS-CoV-2 at real-time PCR (RT-PCR) on nasal swab, were followed up over time to assess the daily number of positive swab tests turning negative (study endpoint). Treatment effectiveness at various time lags since the first positive RT-PCR swab test was measured by rate of events in the experimental arm (EER) and in the control arm (CER), absolute risk increase (ARI) = (EER − CER), and number needed to treat (NNT) = (1/ARI). To investigate the endpoint, we used logistic and Cox regression models, expressing the result as odds ratio (OR) and hazard ratio (HR) with 95% confidence interval (95%CI), respectively. The symptoms recorded with a modified COVID-Q questionnaire at both diagnosis and first negative antigenic swab test were compared in each group (treated versus controls) by exact symmetry test. **Results**: During the first five days of treatment, COVID-19 patients treated with Tonimer Lab Panthexyl 800 were more likely to become negative two days before controls. According to NNT, four subjects had to be treated for five days to achieve the study endpoint in one individual. The negativization rate in patients treated with Tonimer Lab Panthexyl 800 was significantly higher than patients’ treated with standard of care alone (OR = 7.39, 95%CI: 1.83–29.8; HR = 6.12, 95%CI: 1.76–21.32). There was no evidence of side effects. **Conclusions**: Nasal spray with Tonimer Lab Panthexyl 800 was effective against SARS-CoV-2, stopping viral shedding in the treatment arm two days before the control group. This treatment should be continued for at least five days after the first positive swab test for SARS-CoV-2.

## 1. Introduction

COVID-19 is an asymptomatic condition in 40% of cases, and most symptomatic patients present with mild/moderate disease not requiring hospitalization or intensive care, especially during the Omicron wave, when the hospitalization rate was estimated to be 0.3% [1].

The main port of entry for SARS-CoV-2 in the human body is the nasal cavity, where the first cells infected by the virus are likely the multi-ciliated cells of the nasopharynx or trachea or the sustentacular cells of the nasal olfactory mucosa [2]. As a result, SARS-CoV-2 can be detected in the nasal cavity of both symptomatic as well as asymptomatic COVID-19 patients, where viral titers are reportedly higher than in the throat [3].

During the Omicron transmission period, the effectiveness of COVID-19 vaccines progressively decreased, with primary as well as recurrent SARS-CoV-2 infections massively surging from December 2021 onward [4,5].

Since SARS-CoV-2 behaves as a “surface virus” in the nose, vaccine-resistant viral strains could arguably be washed off by nasal rinses, thereby reducing nasal viral shedding time (VST) [6,7,8,9]. Nasal irrigation or aerosol by isotonic or hypertonic saline solution is a traditional therapeutic approach for respiratory or nasal inflammation, also featured by prophylactic properties against upper respiratory infections. Post-secondary analysis of the Edinburgh and Lothians Viral Intervention Study (ELVIS), a pilot randomized controlled trial (RCT), reported reduced VST of coronavirus infection in the upper respiratory tract by 2.6 days using hypertonic saline nasal irrigation and gargling [10,11]. Hypertonic saline nasal irrigation with gargling has also been endorsed as a potentially safe and effective intervention against SARS-CoV-2 infection [10,11].

A recent meta-analysis evaluating both isotonic (0.9% wt/vol sodium chloride solution, close to the physiologic salt concentration of the body) and hypertonic (greater than 0.9% wt/vol) saline solutions against all sino-nasal diseases concluded that hypertonic saline rinses with NaCl concentration < 5% were more beneficial than isotonic saline for the management of sinonasal pathologies [12].

The average salinity of undiluted seawater (a hypertonic solution) is about 3.5% wt/vol. Unlike saline, which consists of NaCl dissolved in distilled water, 99% of seawater salinity is due to six components: Cl^−^, Na^+^, SO_4_^2−^, Mg^2+^, Ca^2+^ and K^+^ [13]. By drawing out water from the mucosal cells of the nasal cavity, hypertonic seawater (800 mOsm/kg osmolality) arguably reduces edema of the local mucosa yet augments the hydration of the mucus layer, hence improving the mucociliary clearance (MCC) [14].

A recent narrative review considered 9 clinical studies and case reports, all supporting the use of hypertonic or isotonic solutions in the early stages of SARS-CoV-2 infection [15]. In 8 studies [6,10,11,16,17,18,19,20], the primary outcome was the duration of sinonasal symptoms, including olfactory dysfunction. This approach, however, tends to exclude asymptomatic COVID-19 patients positive for SARS-CoV-2 at RT-PCR. In one out of 9 clinical studies of the former review on the use of hypertonic or isotonic solutions against early infection by SARS-CoV-2, the primary study endpoint was VST, since study subjects were asymptomatic but positive for SARS-CoV-2 at RT-PCR [21].

Although clinical data are still scanty, nasal and oral sprays display a more convenient application for elderly people or those who are unable to rinse/gargle [22]. However, to the best of our knowledge, there were no studies reporting treatments with hypertonic saline given by nasal spray to reduce VST or hasten the relieve of COVID-19 symptoms [15].

In view of the above, the *Regressed Nasal Infectivity and Shedding of SARS-CoV-2 by Achieving Negativization for COVID-19 Earlier* (RE.NA.I.S.S.A.N.C.E.) clinical trial aimed to assess the efficacy of Tonimer Lab Panthexyl 800, an already existing formulation of sterile hypertonic solution spray, to shorten VST of SARS-CoV-2 from the nasal cavity of infected patients

## 2. Methods

### 2.1. Description of the Trial

Using an already existing medication (Tonimer Lab Panthexyl 800), a sterile hypertonic solution manufactured by Ganassini Corporate (Milan, Italy) containing seawater, xylitol, panthenol and lactic acid to be administered by nasal spray, we conducted a prospective open-label controlled clinical trial in one single center, the COVID-19 point of Treviso (Veneto Region, Northeastern Italy), in order to compare the anti-viral activity of the above drug with respect to standard of care against SARS-CoV-2 infection. The qualitative and quantitative formulation of Tonimer Lab Panthexyl 800 can be viewed in Table 1 (information provided by Ganassini Corporate).

The RENAISSANCE clinical trial was approved by the ethical committee of the Local Health Unit N. 2 *Marca Trevigiana* (991/CE) and was registered on Clincaltrial.gov on 14 July 2022 (NCT05458336) [23].

VST was defined as the time between the first positive and first negative (viral shedding cessation) nasal swab test [24]. The onset of sinonasal symptoms and the performance of a nasal diagnostic swab test are events very close in time in the current phase of the pandemic. Therefore, in the present study, SARS-CoV-2 infection was defined as the first positive RT-PCR result in both symptomatic and asymptomatic patients. All individuals positive for PCR nasal swab tests against SARS-CoV-2 self-tested daily during their domiciliary isolation with antigenic rapid tests until obtaining the first negative result, as a marker of viral shedding cessation,

### 2.2. Eligibility Criteria for Participants

The following criteria of inclusion/exclusion were applied to select COVID-19 patients for this study:

- Agreeing to take part within 48 h since testing positive for SARS-CoV-2 at RT-PCR;
- Age >18 years;
- Mild/moderate COVID-19 symptoms or asymptomatic SARS-CoV-2 infection;
- Provision of written informed consent;
- Completion of COVID-19-Q questionnaire (Supplementary File S1) at study entry as well as exit.

### 2.3. Setting and Location Where the Data Were Collected

Patients testing positive for SARS-CoV-2 at RT-PCR, asymptomatic/pre-symptomatic or affected by mild/moderate COVID-19 symptoms not requiring hospitalization were recruited at the COVID-19 hub of Treviso (Northeastern Italy).

### 2.4. The Interventions by Group

COVID-19 patients were broken down into two groups:

- a treatment arm (receiving standard of care plus nasal spray with Tonimer Lab Panthexyl 800); and
- a control arm (receiving standard of care but nasal spray with Tonimer Lab Panthexyl 800).

The recruitment started with patients assigned to the treatment arm, enrolled from 23 February 2022 until 18 March 2022. Patients assigned to the control group were instead enrolled from 16 March 2022 until 30 March 2022.

#### 2.4.1. Both Treatment and Control Groups

After acquiring verbal consent to enroll, the otolaryngology healthcare staff of Ca’ Foncello Hospital of Treviso explained patients in detail the use of the COVID-19 Antigen Rapid Test Device (for nasopharyngeal swabs), evaluating their ability to perform it autonomously. All patients were provided with a set of swabs for self-testing against SARS-CoV-2, to be performed on a daily basis at home during the isolation for COVID-19 until the first negative antigenic test result. Patients were allowed to return the signed informed document by e-mail at a later stage, to have more time to reflect on their decision to take part in this clinical trial. The information/consent form also acceptance to allow the research team to directly access sensitive/clinical data of study participants.

All study participants were asked to complete a modified COVID-Q questionnaire (Supplementary File S1) provided with a QR code, self-reporting their symptoms and comorbidities, both at COVID-19 diagnosis as well as at the first negative antigenic swab test [25]. The questionnaire included three sections:

- Section 1: collecting socio-demographic information;
- Section 2: collecting information on COVID-19 vaccination status (number, date and type of vaccine received), comorbidities (diabetes, COPD, heart disorders; renal disorders, other) and lifestyle habits (smoking, alcohol and oral hygiene);
- Section 3: collecting COVID-19 symptoms, which were described using the internationally validated score Sino-Nasal Outcome Test 22 (SNOT-22) regarding smell, taste function, and therapy followed.

As soon as a negative antigenic test result was obtained, the patient was required to contact the research team for a second interview on COVID-19 symptoms, identical to that administered at study entry.

#### 2.4.2. The Treatment Group

**Figure 1:**
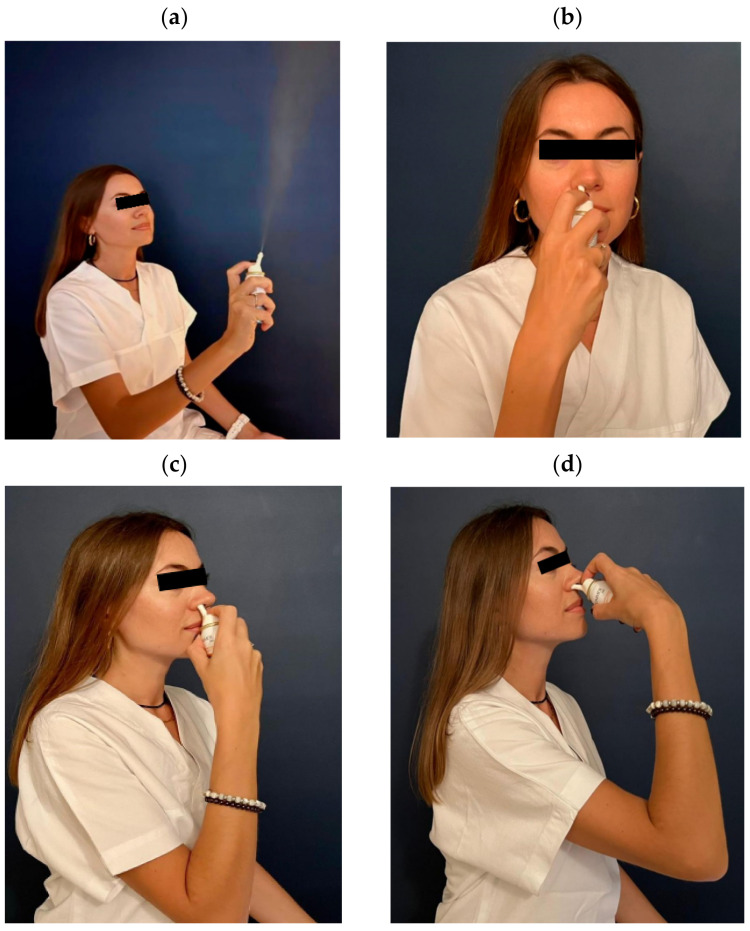
(**a**–**d**) Modality of spraying Tonimer Lab Panthexyl 800 inside the nasal cavity. The patient’s head must be kept in an upright position the intranasal nozzle device is first inserted vertically inside the nostril to penetrate 5 mm into the airspace and subsequently inclined upon the horizontal plane.

Participants assigned to the treatment arm were allowed to take the standard of care, i.e., any medications recommended against COVID-19 plus Tonimer Lab Panthexyl 800 mOsm/Kg (CE 0546), a hypertonic solution based upon seawater enriched with xilitol and panthenol. These subjects were provided with a cylinder used to spray the nasal cavities three times/day for as much as 15 days maximum. Figure 1a–d show the modality to spray Tonimer Lab Panthexyl 800 inside the nasal cavity. As can be seen, the patient’s head must be kept in upright position, the intranasal nozzle device is first inserted vertically inside the nostril to penetrate 5 mm into the airspace and subsequently inclined upon the horizontal plane.

In order to avoid potential false negative test results, patients treated with Tonimer Lab Panthexyl 800 had to self-test against SARS-CoV-2 with an antigenic test before nasal sprays.

### 2.5. Outcome Measure

Treatment effectiveness (study endpoint) was the reduction of VST in treatment as compared to the control arm during the same time-frame of follow-up.

### 2.6. Estimated Sample Size

One nasal spray based on sodium hypochlorite reportedly reduced viral titers of SARS-CoV-2 in vitro by more than two orders of magnitude [22]. We hypothesized that such inactivation in vivo could be 10 times. In the present trial measuring dichotomous events (positive swab test: no versus yes), the total sample size (N) required to detect an experimental-group proportion of 0.2 when the control-group proportion was 0.02, assuming a two-sided hypothesis test with a 5% significance level and a desired power of 80%, was 94 (47 per group, when both groups had the same number of observations).

### 2.7. Statistical Analysis

The main characteristics of the two study groups, all expressed as categorical variables, were compared with the chi-squared (χ^2^) test.

In this prospective clinical trial two groups of subjects (treated and controls), both testing initially positive for SARS-CoV-2 at RT-PCR on nasal swabs, were followed over time to ascertain the daily number of positive swabs turning negative in each group. The effectiveness of the treatment was derived from the following two proportions:

- **Rate of events in the experimental arm (EER)** = number of events/number of patients in the experimental arm);
- **Rate of events in the control arm (CER)** = number of events/number of patients in the control arm.

Using the above data, and given that treatment was beneficial (stopping the viral shedding), the clinical significance measures were calculated as follows [26]:

- **Absolute risk increase (ARI) = (EER − CER).** It expresses, generally in decimal values, the absolute increase in the risk of events in the treated group compared to controls. The sign of ARI is positive when EER > CER and negative otherwise.
- **Number needed to treat (NNT) = (1/ARI).** NNT represents the expected number of patients required in order to achieve one beneficial outcome event. This estimate is easy to calculate and expresses the benefits of an intervention in the same unit of measurement (number of patients).

In this clinical trial measuring a dichotomous endpoint, the statistical significance of treatment effectiveness was assessed by using the following analyses:

- Logistic regression, expressing the results as odds ratio (OR) with a 95% confidence interval (95%CI). OR is the ratio of the probability of the event in the treatment arm against the probability of the event in the control group. It is expressed in decimal values. OR > 1.00 or < 1.00 expresses, respectively, a beneficial or a detrimental effect of the treatment.
- Cox regression (or proportional hazards regression), expressing the results as hazard ratio (HR) with a 95% confidence interval (95%CI). HR investigates the effect of the treatment on the time until first negative antigenic swab test result. Since this was a beneficial intervention (because treatment stopped viral shedding), a positive HR indicates a protective effect of the associated variable. Cox regression allows the investigation of the effect of multiple variables at the same time. Since all terms displayed in Table 2 were not significant in univariable Cox proportional hazard regression, the analysis was restricted to estimate the treatment effect.

Intention to treat (ITT) analysis was adopted. ITT analysis includes all randomized patients in the groups to which they were allocated, regardless of their adherence to the entry criteria, the treatment received and any subsequent deviation from the study protocol [27].

The symptoms recorded with a modified COVID-Q questionnaire at both COVID-19 diagnosis and first negative antigenic test were compared to each other using the exact symmetry test (which for 2 × 2 tables are reduced to an exact McNemar test). The presence of one group that received the intervention (treatment group) and one group that did not (control group) allows the separation of the effect of the intervention from that of other circumstances.

Stata 14.2 (Stata Corporation, College Station, TX, USA) was employed for the analysis.

## 3. Results

One hundred twenty subjects testing positive for SARS-CoV-2 (all infected by the Omicron variant) were consecutively recruited from the COVID-19 center of Treviso between 23 February 2022 through 30 March 2022. Twelve subjects had to be dropped from the analysis: three in the treatment arm for inconsistency of dates of the swab test; 7 in the experimental arm and two in the control arm because of failing to return the signed informed consent form after initial verbal agreement. The final number of study participants included 50 patients treated with Tonimer Lab Panthexyl 800 against 58 controls, for a total number of 108 study participants.

Table 2 displays the distribution of variables collected. The number of females (N = 65) was slightly higher than males (N = 43), and 87% (=94/108) of subjects were younger than 60 years. The vast majority were non-smokers (72.2% = 78/108) or ex-smokers (14.8%=16/108). Most participants were occasional drinkers (58.3%=63/108) or non-drinkers (36.1%=39/108). Ninety-two patients (85.2%) had received three doses of the COVID-19 vaccine at the time of entering the study, and 5.6% (=6/108) were unvaccinated against SARS-CoV-2. For the first two doses, Comirnaty (Pfizer-Biontech) was by far the most used vaccine, whereas for the third dose 59.3% (=54/91) patients had received Spikevax (Moderna) and 40.7% (=37/91) Comirnaty. In terms of pre-existing medical conditions, 17.6% (=18/102) patients had hypertension, 1.9% (=2/108) diabetes, 0.9% (=1/108) cerebrovascular disease, 6.5% (7/108) cancer, 13.0% (=14/108) had chronic obstructive pulmonary disease (COPD) and 0.9% (=1/108) renal failure. As can be seen in Table 2, there was no statistical difference between the treatment and control arms for any variable.

Table 3 shows, by VST (expressed as number of days elapsed between the first PCR positive and first negative antigenic swab test for SARS-CoV-2; notice that “12+” includes days 12, 13, 14 and 15, summed up together since subjects were few) the following quantities:

- Number of negative antigenic swab test results (study endpoint) in treatment versus control arm;
- Proportion of endpoint in the experimental (experimental event rate (EER)) versus control (control event rate (CER)) group;
- Absolute risk increase (ARI), computed as the difference between EER and CER.

The lack of negative antigenic swab test results on days 2 to 4 in the control group is noteworthy. Using day 6 as the cutoff point because EER ≅ CER, it can be seen that ARI presents only positive results from day 2 to day 5, suggesting that the endpoint (negative antigenic swab test result) in the treatment arm was more common during the first days of Tonimer Lab Panthexyl 800 application.

**Figure 2:**
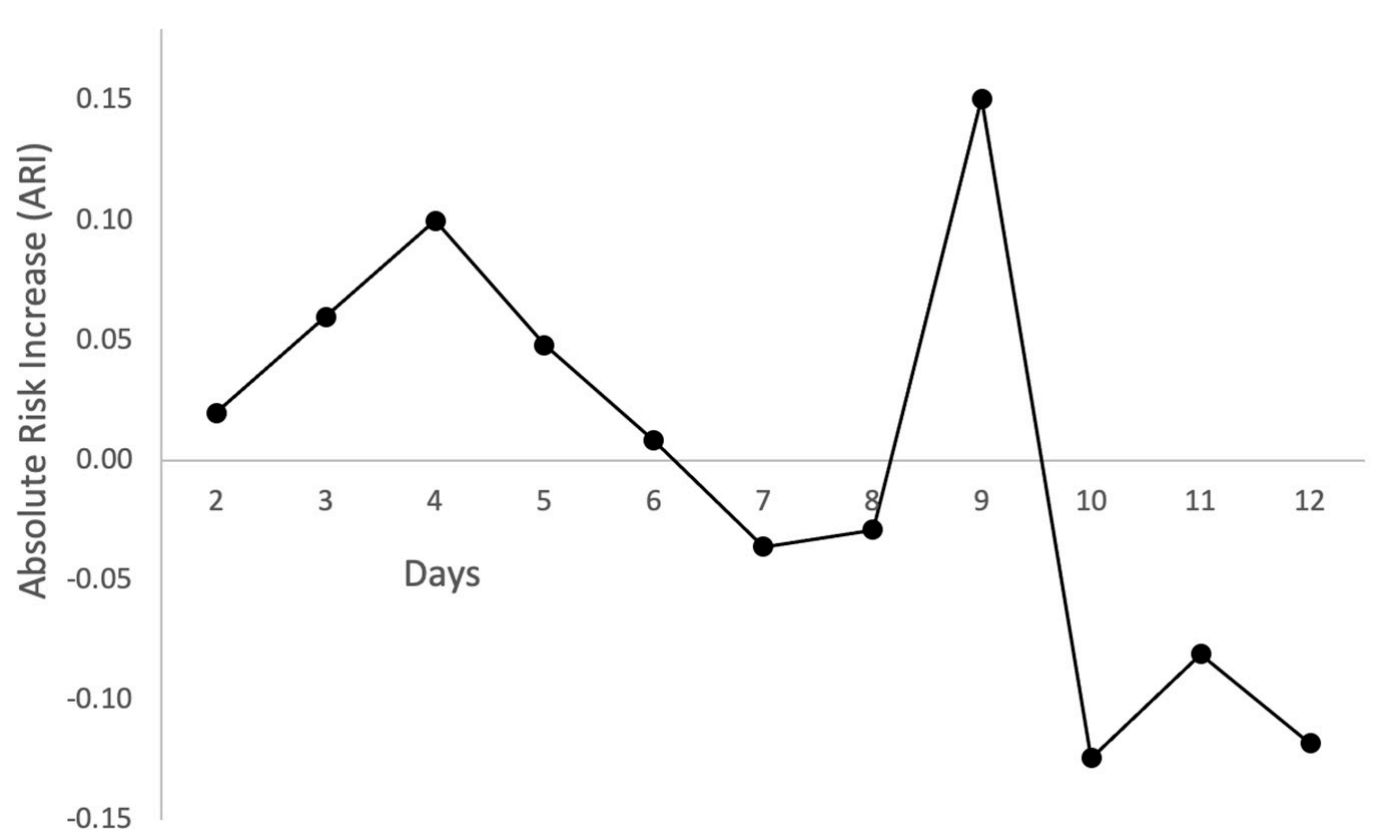
Distribution of absolute risk increase (ARI) over time (days between initial positive and first negative SARS-CoV-2 swab test result).

**Figure 3:**
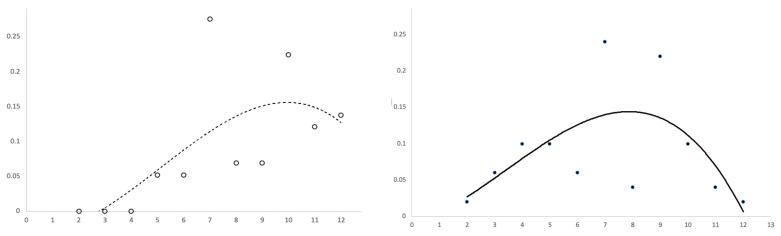
Cartesian scatter plots reporting control event rate (CER, left plot) or experimental event rate (EER, right plot) on the vertical axis, by days since initial positive and first negative swab test result against SARS-CoV-2 (horizontal axis). The two (dotted and continuous) lines represent a third degree polynomial function.

**Figure 4:**
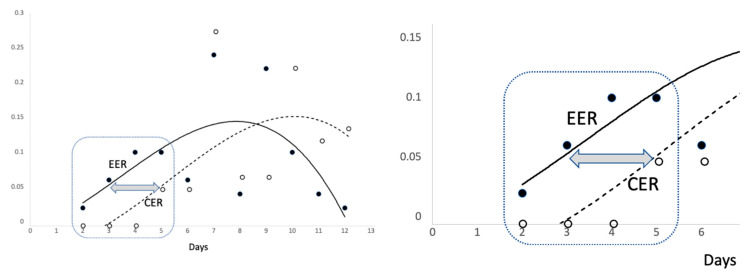
Experimental event rate (EER) and control event rate (CER) by days since initial positive and first negative SARS-CoV-2 swab test result. The two (dotted and continuous) lines represent a polynomial function of the third degree.

Figure 2, Figure 3 and Figure 4 show several scatter plots using Cartesian coordinates to display the values for two variables from the set of data in Table 3.

Figure 2 displays days on the horizontal axis and ARI on the vertical axis. The trend is clearly upward at the beginning of the plot, peaking at day 4, declining afterward until day 6 and fluctuating from day 7 onward.

Figure 3 shows two scatter plots reporting days on the horizontal axis and CER (left plot) or EER (right plot) on the vertical axis. Since data contained many fluctuations, a curved trend line was fitted using a third degree polynomial function. CER and EER points were reasonably close to the trend line in the first (left) part of each graph, while from day 6 onward the points became over-dispersed.

CER and EER points and trend lines were coded using different signs. Therefore, the two plots of Figure 3 were overlapped in Figure 4 so that both images can be visualized. The scattergram of Figure 4 can be broken down into two parts.

The encircled image (first part of the scatter plot) has been magnified to better visualize some details. As can be seen, the curved trend appears linear and the sets of points (EER and CER) cluster together closely. Interestingly, the horizontal distance between the red and blue line is of about two days. Therefore, with respect to controls, subjects treated with Tonimer Lab Panthexyl 800 nasal spray achieved negativization of nasal swab test for SARS-CoV-2 approximately two days earlier than controls.

Table 3 also shows the rate of events in the experimental versus control group along with estimates and 95% confidence intervals for ARI, NNT and OR in two time blocks: from day 2 to day 5 and from day 7 to day 15 since positive RT-PCR swab test for SARS-CoV-2. In the first block, ARI had a positive sign and OR was significantly above unity, indicating a beneficial effect of treatment. According to NNT, four subjects had to be treated for 5 days to achieve a VST reduction in one individual. In these subjects, the negativization of the antigenic swab test was attained two days earlier than that in the control group (Figure 3). By contrast, the treatment had a detrimental effect in the second time block, as shown by the negative sign of ARI and OR being lower than 1.00. In this block of time, NNT (being equal to 1/ARI, namely 1/−0.24) was not estimated since it would have a negative sign.

The hazard ratio (HR) was 6.12 with a 95% confidence interval ranging from 1.76 to 21.32 and *p* = 0.004 in the Cox proportional hazard regression model, confirming that the new treatment during the first 5 days since first positive PCR test for SARS-CoV-2 was significantly more effective than the standard of care alone in reducing nasal VST.

Table 4 shows several symptoms (of anosmia, allergy, and irritation of nasal, oral or pharyngeal mucosa) and medicines commonly used to treat these conditions. For each one, the columns of Table 4 display the number of subjects with or without the symptom, recorded in the questionnaire at study entry and exit, along with the exact significance probability in both the treatment and control arms. Considering “*Dry cough*”, in the treatment group the number of subjects without the symptom was 18 at study entry and 36 at exit, a highly significant difference (*p* < 0.001). Likewise, in the control group, 23 subjects without dry cough before the study became 42 after the end of the study, resulting in a highly significant difference (*p* < 0.001). The parallel common trend in both groups suggests a natural change towards a normal state of health. The latter pattern was observed most often. Considering “*Productive cough*”, the differences between study entry and exit were not significant but figures still suggested an improvement. There was no evidence of adverse effects, e.g., before–after deterioration in the treatment but in the control arm.

Furthermore, apart from a few symptoms improving more in controls (productive cough, stuffy nose, runny nose, breath shortage, anosmia/ageusia), most conditions (ear wadding, dry cough, lacrimation, feverish sensation, sweating, shivering, headache, sore throat, aching sinusitis) waned more frequently in the treatment arm, which was also characterized by reduced use of anti-pyretic as well as nasal spray medications (Table 4).

## 4. Discussion

### 4.1. Key Findings

COVID-19 patients treated with Tonimer Lab Panthexyl 800 were more likely to become negative two days before controls during the first five days of treatment. NNT analysis indicated that four subjects had to be treated for five days to achieve a reduction in VST in one patient (Table 3). Beneficial effects were observed for treatments lasting up to five days, vanishing from day 7 onward.

Since symptoms did not increase with treatment, no adverse effects could be attributable to Tonimer Lab Panthexyl 800. Moreover, improvement of most symptoms was stronger in the treatment than in control arm.

### 4.2. Limitations

Twelve individuals were excluded from the analysis after being assigned to their study arm: 9 patients failing to return the signed informed consent and three incurring a protocol violation, since positive and negative swab test results occurred on the same day of recruitment. In such cases, patients can be excluded from ITT analysis [28].

We had an unequal number of observations. Nonetheless, the estimated power was 0.9081 using a two-sided test to detect an experimental-group proportion of 0.28 (observations = 50) against the control-group proportion of 0.05 (observations = 58) assuming a 5% significance level.

Finally, information on time since the onset of the first symptom (a potential confounder in the analysis) was not available. However, COVID-19 presents with a rather variable clinical spectrum—especially among patients affected by mild/moderate disease—with a high proportion of asymptomatic patients, hence information on symptom onset is inevitably prone to recall bias.

Although the procedure for using the medical device was extensively explained, patients were not directly observed during the self-administration of Tonimer Lab Panthexyl 800 spray at home. In order to maximize virucidal efficacy, topical formulations against respiratory viruses should target the nasopharynx, the main infection settlement for SARS-CoV-2. If the nozzle of the medical device is inserted in the nose almost vertically, the deposition of drug droplets in the nasopharynx may be suboptimal. Therefore, after an initial vertical insertion to the top of the nostril, the medical device should be bent horizontally slightly toward the cheeks, to penetrate 5 mm into the nasal airspace.

Information on PCR cycle threshold (CT) would have been more accurate to assess the nasopharyngeal negativization than antigenic test. Nonetheless, in a subset of 60 patients (34 treated with Tonimer and 26 controls) randomly selected, a negative antigenic test result was followed by a confirmatory RT-PCR, showing full concordance between the two tests.

Although specific information on SARS-CoV-2 sub-lineages infecting each patient was not available, only Omicron BA.2 was known to be circulating in Treviso area during the study period, and hence we do not expect any difference in viral sub-variants between patients treated with Tonimer Lab Panthexyl 800 and controls. Moreover, the resulting molecule HOCl is highly reactive and highly virucidal when dissolved in water, regardless of the change in amino acids of the viral proteins. Given evidence from clinical studies, HOCl dissolved in water is still recommended for hand washing by the United States Environmental Protection Agency (USEPA) as an effective disinfectant against COVID-19, notwithstanding the continuous emergences of new SARS-CoV-2 sub-lineages [29].

### 4.3. Interpretations of Findings

The most relevant pharmacological effects of hypertonic saline/seawater against SARS-CoV-2 are the following [30]:

- *Inhibition of viral replication*. SARS-CoV-2 replication is reportedly dose-dependently inhibited by saline solutions (0.8–1.7% NaCl). Inhibition of viral replication already started from a concentration of 0.6%, increasing up to 50% at 0.9% (isotonic saline solution) and reaching 100% at 1.5% (mildly hypertonic saline solution) [31]. Saline, however, had no direct effect on SARS-CoV-2 itself. Inhibition of viral replication in vitro was arguably due to an intracellular mechanism of membrane depolarization and intracellular energy deprivation efficiently stimulated by hypertonic saline solutions [31].
- *Shift of myeloperoxidase (MPO) activity in epithelial or phagocytic cells*. This metabolic route yields hypochlorous acid (HOCl). Inhibition of viral replication in presence of chloride and halide salts was first reported in the 1960s [32]. HOCl has the well-known virucidal activity of bleach, effective against all viruses. Nevertheless, HOCl is also cytotoxic and may injure the epithelial cells of human airways. For instance, direct exposure of mucosal cilia to HOCl was found to cause ciliostasis, possibly contributing to discrepant effects of hypertonic saline solutions on the MCC across different studies. Therefore, tight regulation of this metabolic route is recommended [33]. Significantly higher expressions of MPO (≈4 fold, *p* < 0.05) were found in naso-oropharyngeal samples of SARS-CoV-2 patients. Over-expression of MPO may produce HOCl in excess, thereby damaging nasopharyngeal tissues [34].

The first part of the results of the present study likely agrees with both the above mechanistic hypotheses. Nasal spray with seawater and other additives achieves an early negativization of an initial positive swab test against SARS-CoV-2 through inhibition of viral replication [31] and shift of MPO activity in epithelial or phagocytic cells producing HOCl [30]. Both mechanisms are stimulated by the supply of chlorine, which was directly conveyed by Tonimer Lab Panthexyl 800 nasal spray in the experimental arm, while it was physiologically generated by the nasal epithelial cells after a time-lag of about three days in control subjects (see CER in Table 3 and Figure 2 and Figure 3).

There were no similar findings for comparison.

The second part of the results, exhibiting no apparent pattern, could be interpreted as a “*dynamical system*” that is simply a function of possible outputs which can also be inputs [35]. It is well known that HOCl is also cytotoxic and may injure the epithelial cells of human airways. In fact, direct exposure of mucosal cilia to HOCl reportedly causes ciliostasis [34], possibly contributing to discrepant effects of hypertonic saline solutions on MCC across different studies. Mucosal areas with cilia damaged by exposure to HOCl may be at higher risk of re-infection, inducing a vicious cycle with detrimental implications. For this reason, tight regulation of this metabolic route has been recommended [34].

For this purpose, we suggest a formulation including either thiocyanate (also known as anion SCN^−^) or the amino acid taurine or both substances to a hypertonic saline solution. The highly reactive molecule HOCl, which also exhibits promiscuous reaction chemistry (i.e., it is highly toxic), oxidizes SCN^−^ into OSCN^−^ (hypothiocyanite) and, separately, taurine into N-chlorotaurine (NCT) [36]. These products are part of the natural protective system of human airways against pathogen threats [7]. The lack of these metabolic routes in human nasal mucosae may explain the survival and proliferation of bacteria and respiratory viruses in the nasal cavity and their subsequent shedding in the environment [37]. Both products—OSCN^−^ and NCT—are less reactive but more selective (i.e., less toxic) than HOCl. For example, OSCN^−^ oxidized the thiol moiety R–SH essential for the activity of numerous enzymes and proteins, thereby inhibiting bacterial glycolysis, respiration, and glucose transport. A recent in vitro study showed that micromolar concentrations of OSCN^−^ exhibited dose- and time-dependent virucidal activity against SARS-CoV-2, without toxicity on Vero cells [9]. On the other hand, NCT was proven to have cidal activity against bacteria, fungi, parasites, and viruses (herpes simplex virus type 1, herpes simplex virus type 2, adenovirus, influenza virus A and HIV-1) and could be applied to sensitive body regions as an endogenous antiseptic. The ciliary beat frequency of epithelial cells of the nasal mucosa, a very sensitive parameter for toxicity, was decreased only moderately and reversibly following exposure to 1% NCT [38].

In the present study, we employed an already existing treatment including xylitol and panthenol as excipients of the hypertonic saline solution. Xylitol is a naturally occurring chemical compound with inherent antimicrobial properties. A systematic review was recently conducted on five RCTs where xylitol was compared to a nasal saline additive for the treatment of sinonasal disease, using as the primary outcome the difference in the 22-item Sinonasal Outcome Test (SNOT-22). Compared to a predefined Minimal Clinically Important Difference score of 8.9, the post-surgical Endoscopic Sinus Surgery (ESS) subgroup met this threshold, whereas the non-surgical subgroup failed to meet the threshold. In conclusion, xylitol may be an effective agent for the treatment of sinonasal disease in post-surgical ESS patients [39]. On the other hand, panthenol has emerged in the field of dermatology and skin care to prevent skin irritation and stimulate skin regeneration and wound healing. Although discovered decades ago, the exact mechanisms of action of panthenol have not been fully elucidated yet [40]. There are no studies on the activity of panthenol or xylitol against SARS-CoV-2 or other viruses; since these two substances may not be useful, they may be removed from an intranasal formulation against SARS-CoV-2.

### 4.4. Prospects

Our data support the suggestion to consider five days of treatment in future clinical trials [31]. This decision was derived from the following considerations:

- Once a person is infected, the median “latent period”, preceding the communicability window of SARS-CoV-2 infection, is ≈3 days followed by ≈4 days of close to maximal infectiousness [41,42].
- According to the standard behavior of upper respiratory viruses, if symptoms deteriorate in five days after disease onset or persist beyond 10 days, it is likely that there is a secondary bacterial infection requiring clinical evaluation [43].
- The UK Health Security Agency allows healthcare workers (HCWs) to return to work if they test negative on both days 5 and 6 (undertaken 24 h apart) after the date of the initial positive RT-PCR swab test [44].
- Current occupational guidelines by the European Centre for Disease Prevention and Control (ECDC) for HCWs testing positive for SARS-CoV-2 recommend a standard isolation of six days for vaccinated versus 10 days for individuals unvaccinated against COVID-19 [45].

If confirmed in further clinical trials, the present findings could support the reduction of the communicability window of SARS-CoV-2 infection by 2–3 days in the community. This intervention could also arguably support a return to work of asymptomatic individuals from COVID-19 isolation 2–3 days earlier.

Since the communicability of SARS-CoV-2 is considerable [14], especially during the Omicron transmission period [46], patients should observe good hand hygiene and decontaminate the surrounding fomites (e.g., sink, counters) and plastic bottles to prevent contact-induced infections. Tackling the presence of viral load on inanimate surfaces is critical because SARS-CoV-2 is reportedly stable on plastic materials, where it can be detected >72 h after fomite contamination [47].

## 5. Conclusions

Nasal spray with Tonimer Lab Panthexyl 800 was effective against SARS-CoV-2, stopping viral shedding in the treatment arm two days earlier than controls. No evidence of adverse effects was found. The treatment should be continued for at least five days, since there is no contraindication to extend it beyond.

## Figures and Tables

**Table 1:** Qualitative–quantitative formulation of Tonimer Lab Panthexyl 800 nasal spray. w/w = weight/weight; q.s. = quantum sufficit.

Components	% w/w
Panthenol	5.0
Xylitol	5.0
Seawater (Maris Aqua)	10.0
Lactic acid 90%	q.s. pH 4.8–5.0
Purified water	q.s. 100%
**Total**	**100.00**

**Table 2:** Main characteristics of study participants, by treatment and control group. Number (N), column percentage (%) and *p*-value (chi-square test).

Variables		Categories	Treated (N = 50) N (%)	Controls (N = 58) N (%)	*p*-Value
**Sex**		**Female**	30 (60.0)	35 (60.3)	0.971
		**Male**	20 (40.0)	23 (39.7)	
**Age (years)**		**<40**	19 (42.2)	22 (38.6)	0.933
		**40–59**	20 (44.4)	27 (47.4)	
		**60+**	6 (13.3)	8 (14.0)	
**Smoking** **status**		**Non-smoker**	36 (72.0)	42 (72.4)	0.530
		**Ex-smoker**	9 (18.0)	7 (12.1)	
		**Current smoker**	5 (10.0)	9 (15.5)	
**Alcohol** **consumption**		**Non-drinker**	20 (40.0)	19 (32.8)	0.285
		**Occasional drinker**	29 (58.0)	34 (58.6)	
		**Regular drinker**	1 (2.0)	5 (8.6)	
**Number of doses of COVID-19 vaccines**		**0**	5 (10.0)	1 (1.7)	0.148
		**1**	1 (2.0)	0	
		**2**	5 (10.0)	4 (6.9)	
		**3**	39 (78.0)	53 (91.4)	
**Vaccine type**	**1st** **dose**	**Comirnaty**	36 (80.0)	47 (82.5)	0.480
		**Spikevax**	6 (13.3)	4 (7.0)	
		**Vaxzevria**	3 (6.7)	6 (10.5)	
	**2nd** **dose**	**Comirnaty**	35 (80.0)	47 (82.5)	0.710
		**Spikevax**	6 (13.6)	5 (8.8)	
		**Vaxzevria**	3 (6.8)	5 (8.8)	
	**3rd** **dose**	**Comirnaty**	18 (46.2)	19 (36.5)	0.355
		**Spikevax**	21 (53.9)	33 (63.5)	
		**Vaxzevria**	0	0	
**Hypertension** **(missing: 6)**		**No**	37 (82.2)	47 (82.5)	0.975
		**Yes**	8 (17.8)	10 (17.5)	
**Diabetes**		**No**	49 (98.0)	57 (98.3)	0.916
		**Yes**	1 (1.8)	1 (1.7)	
**Cardiovascular** **diseases**		**No**	50 (100)	58 (100)	NA
		**Yes**	0	0	
**Cerebrovascular diseases**		**No**	49 (98.0)	58 (100)	0.279
		**Yes**	1 (1.8)	0	
**Cancer**		**No**	47 (94.0)	54 (93.1)	0.850
		**Yes**	3 (5.3)	4 (6.9)	
**COPD**		**No**	42 (84.0)	52 (89.7)	0.383
		**Yes**	8 (14.0)	6 (10.3)	
**Renal disease**		**No**	50 (100)	57 (98.3)	0.351
		**Yes**	0	1 (1.7)	

**Table 3:** Number of negative antigenic swab test results (=study endpoint) in treatment versus control arm, by number of days since initial positive RT-PCR swab test for SARS-CoV-2; proportion of endpoint in the experimental (experimental event rate (EER)) and control (control event rate (CER)) group; absolute risk increase (ARI), computed as difference between EER and CER since treatment increases the risk of endpoint. NNT = number needed to treat. Rate of events in the experimental (EER) and control (CER) arm, by two time blocks (days 1–5 versus days 7–15) with estimates and 95% confidence intervals (95%CIs) of four measures of clinical significance: absolute risk increase (ARI), number needed to treat (NNT), odds ratio (OR) and hazard ratio (HR).

Day	Treated	Controls	EER		CER		ARI		NNT (95%CI)	OR (95%CI)	HR (95%CI)
			Daily	Pooled	Daily	Pooled	Daily	Pooled (95%CI)			
2	1	0	0.02	0.28	0	0.05	0.02	0.23 (0.10; 0.36)	4 (3;10)	7.39 (1.83; 29.8)	6.12 (1.76; 21.32)
3	3	0	0.06		0		0.06				
4	5	0	0.1		0		0.10				
5	5	3	0.1		0.05		0.05				
6	3	3	0.06		0.05		0.01				
7	12	16	0.24	0.66	0.28	0.89	−0.04	−0.24 (−0.09; −0.38)	NA	0.22 (0.08; 0.65)	NA
8	2	4	0.04		0.07		−0.03				
9	11	4	0.22		0.07		0.15				
10	5	13	0.10		0.22		−0.12				
11	2	7	0.04		0.12		−0.08				
12 +	1	8	0.02		0.14		−0.12				
**Total**	**50**	**58**									

**Table 4:** Number of study participants by symptoms at initial positive (“before”) and first negative (“after”) swab test for SARS-CoV-2, with exact significance probability (*p*-value) in both arms (treatment versus control).

Symptoms and Use of Medicines		Treatment Arm (N = 50)			Control Arm (N = 58)		
		Before	After	*p*-Value	Before	After	*p*-Value
**Dry cough**	**No**	18	36	<0.001	23	42	<0.001
	**Yes**	21	2		19	13	
**Productive cough**	**No**	30	37	0.119	30	41	0.047
	**Yes**	15	13		12	8	
**Ear wadding**	**No**	31	41	0.029	30	45	0.003
	**Yes**	45	13		12	7	
**Stuffy nose**	**No**	14	32	<0.001	13	28	0.001
	**Yes**	21	24		22	14	
**Runny nose**	**No**	13	31	<0.001	18	36	<0.001
	**Yes**	24	16		24	13	
**Frequent** **sneezing**	**No**	18	39	<0.001	26	44	<0.001
	**Yes**	22	10		22	10	
**Anosmia/ageusia**	**No**	37	39	0.662	41	43	0.710
	**Yes**	7	10		7	6	
**Lacrimation**	**No**	37	41	0.508	43	54	0.006
	**Yes**	12	3		7	6	
**Hoarseness**	**No**	16	36	<0.001	15	40	<0.001
	**Yes**	25	18		24	10	
**Feverish sensation**	**No**	20	43	<0.001	20	53	<0.001
	**Yes**	34	4		25	5	
**Sweating**	**No**	37	43	0.227	40	52	0.006
	**Yes**	16	2		9	4	
**Shivering**	**No**	29	46	<0.001	37	54	0.002
	**Yes**	20	1		18	3	
**Headache**	**No**	22	38	<0.001	21	47	<0.001
	**Yes**	27	10		17	11	
**Throat** **discomfort**	**No**	25	44	<0.001	16	44	<0.001
	**Yes**	30	12		23	7	
**Sore throat**	**No**	27	35	<0.001	20	50	<0.001
	**Yes**	31	7		20	5	
**Aching sinusitis**	**No**	27	35	0.083	27	44	0.002
	**Yes**	21	10		12	11	
**Air hunger**	**No**	42	47	0.063	50	54	0.219
	**Yes**	5	3		5	3	
**Breath shortage**	**No**	33	40	0.119	43	45	0.712
	**Yes**	9	6		11	6	
**Anti-pyretic** **medications**	**No**	27	32	0.323	26	42	0.001
	**Yes**	18	14		8	15	
**Penicillin intake**	**No**	47	45	1.000	57	58	1.000
	**Yes**	1	0		1	2	
**Inhaling spray for COPD**	**No**	46	48	1.000	54	56	0.500
	**Yes**	2	2		2	2	
**Tablets for COPD**	**No**	46	50	0.125	56	56	1.000
	**Yes**	1	1		4	0	
**Antitussive drugs**	**No**	38	35	0.631	39	40	0.071
	**Yes**	6	13		3	9	
**Eye drop use**	**No**	49	50	1.000	54	56	1.000
	**Yes**	2	2		1	0	
**Nasal spray** **medications**	**No**	44	10	0.180	24	25	<0.001
	**Yes**	4	0		0	1	

## Data Availability

The datasets generated and analysed during the current study are not publicly available, since they were purposively collected by the authors for the present study. They may be available from the corresponding author on reasonable request.

## Supplementary Materials

The following supporting information can be downloaded at: https://www.mdpi.com/article/10.3390/pharmaceutics14112502/s1, File S1: COVID-19 Questionnaire.
