# Supplementary material for: Early Negativization of SARS-CoV-2 Infection by Nasal Spray of Seawater plus Additives: The RENAISSANCE Open-Label Controlled Clinical Trial
Source: Pharmaceutics. 2022 Nov 18;14(11):2502. doi: 10.3390/pharmaceutics14112502 (PMC9696981; doi:10.3390/pharmaceutics14112502)
Supplement: Supplementary file 1 [file pharmaceutics-14-02502-s001.zip › pharmaceutics-1984873-supplementary.pdf]

Supplemetary Fle (S1). COVID-19 QUESTIONNARIRE

**SECTION 1: PERSONAL INFORMATION**	
Date of birth	
Date of first nasal positive swab test for SARS-CoV-2	
Date of first nasal negative swab test for SARS-CoV-2	
Why were you tested for COVID-19 ?	COVID 19 related symptoms
	close contact with a positive subject
	Both
Did you have COVID-19 before?	No
	Yes
If you answered yes before, when did it happens and how many times?	
How many COVID-19 vaccine doses did you received? (please specify also date of vaccinations and vaccine type)	1
	2
	3
	None
**SECTION 2: COMORBIDITIES, VACCINATION STATUS AND DENTAL HYGIENE**	
Sex	Female
	Male
Smoking	Never smoker
	Ex smoker
	Current smoker
Alcohol consumption	No
	Occasionally
	Yes

Do you have high blood pressure?	No
	Yes
	I take medications for it
Do you suffer from diabetes?	No
	Yes
Do/Did you suffer from cardiovascular disorders (including heart rate disorders)	No
	Yes
Do/Did you suffer from cerebrovascular disorders (stroke, TIA, other)?	No
	Yes
Do/did you have cancer?	No
	Yes
Do you suffer from chronic obstructive pulmonary disease (COPD)?	No
	Yes
Are you in renal failure?	No
	Yes
Do you suffer of liver failure?	No
	Yes
Do you brush your teeth?	No
	Yes
How many time do you brush your teeth every day?	
**SECTION 3: COVID-19 SYMPTOMS**	
Do you have now dry cough?	No
	Yes, to some extent
	Yes, a lot

Do you have now productive cough?	No
	Yes, to some extent
	Yes, a lot
Do you have now a sensation of a blocked ears?	No
	Yes, to some extent
	Yes, a lot
Do you have now a sensation of stuffy nose?	No
	Yes, to some extent
	Yes, a lot
Do you have now a sensation of running nose?	No
	Yes, to some extent
	Yes, a lot
Do you sneeze now frequently?	No
	Yes, to some extent
	Yes, a lot
Do you now have now lacrimation?	No
	Yes, to some extent
	Yes, a lot
Do you have now hoarseness or lump in your throat?	No
	Yes, to some extent
	Yes, a lot
Do you have now fever?	No
	Yes, to some extent
	Yes, a lot

Do you now sweat?	No
	Yes, to some extent
	Yes, a lot
Do you feel now chilly?	No
	Yes, to some extent
	Yes, a lot
Do you feel now headache?	No
	Yes, to some extent
	Yes, a lot
Do you have now throat discomfort?	No
	Yes, to some extent
	Yes, a lot
Do you have sore throat?	No
	Yes, to some extent
	Yes, a lot
Do you have muscle ache?	No
	Yes, to some extent
	Yes, a lot
Do you feel joint pain?	No
	Yes, to some extent
	Yes, a lot
Do you feel thoracic pain?	No
	Yes, to some extent
	Yes, a lot

Do you have sinusitis pain (a sensation of blocked nose, pressure on your face, frontal headache)?	No
	Yes, to some extent
	Yes, a lot
Do you have nek swelling?	No
	Yes, to some extent
	Yes, a lot
Have you lost your appetite?	No
	Yes, to some extent
	Yes, a lot
Do you have respiratory problems?	No
	Yes, to some extent
	Yes, a lot
Do you have now dyspnea?	No
	Yes, to some extent
	Yes, a lot
Do you feel shortness of breath when you walk?	No
	Yes, to some extent
	Yes, a lot
Can you think straight?	No
	Yes, to some extent
	Yes, a lot
Do you have difficulties with your daily routine?	No
	Yes, to some extent
	Yes, a lot

Do you feel lazy?	No
	Yes, to some extent
	Yes, a lot
Do you feel tired?	No
	Yes, to some extent
	Yes, a lot
Do you feel different?	No
	Yes, to some extent
	Yes, a lot
Do you feel so bad that you must stay in bed?	No
	Yes, to some extent
	Yes, a lot
Do you have poor sleep quality?	No
	Yes, to some extent
	Yes, a lot
Do you wake up often at night?	No
	Yes, to some extent
	Yes, a lot
Do you find it difficult to fall asleep?	No
	Yes, to some extent
	Yes, a lot
Are you awake most of the night?	No
	Yes, to some extent
	Yes, a lot
Do you feeling moody?	No
	Yes, to some extent
	Yes, a lot

Do you feel irritable?	No
	Yes, to some extent
	Yes, a lot
Are you taking antipiretic medications?	No
	Yes, to some extent
	Yes, a lot
Are you taking painkillers?	No
	Yes, to some extent
	Yes, a lot
Are you taking antibiotics?	No
	Yes, to some extent
	Yes, a lot
Are you taking you take spray or inhalers for asthma or chronic obstructive bronchopneumonia?	No
	Yes, to some extent
	Yes, a lot
Are you taking tablets for asthma or chronic obstructive bronchopneumonia?	No
	Yes, to some extent
	Yes, a lot
Are you taking you take anti-tussive medications?	No
	Yes, to some extent
	Yes, a lot
Are you taking you taking eye drops?	No
	Yes, to some extent
	Yes, a lot

Are you taking you take nasal spray?	No<br>Yes, to some extent<br>Yes, a lot
Do/Did you have diarrhoea?	No<br>Yes, to some extent<br>Yes, a lot
Do you feel nausea?	No<br>Yes, to some extent<br>Yes, a lot
Do/did you have vomit?	No<br>Yes, to some extent<br>Yes, a lot
Do you feel stomach-ache?	No<br>Yes, to some extent<br>Yes, a lot
Do you feel dizzy?	No<br>To some extent<br>Yes, a lot
Do you feel anosmia/ageusia<br>(reduced/altered sense of taste and/or smell)?	No<br>Yes, marginally<br>Yes, to some extent<br>Yes, moderate<br>Yes, a lot<br>Yes, completely
Do you have any other symptoms that we haven’t asked for?<br>(please specify)	
